# Monitoring and Evaluating Progress towards Universal Health Coverage in Ethiopia

**DOI:** 10.1371/journal.pmed.1001696

**Published:** 2014-09-22

**Authors:** Abebe Alebachew, Laurel Hatt, Matthew Kukla

**Affiliations:** 1 Breakthrough International Consult, Addis Ababa, Ethiopia; 2 Health Finance and Governance Project, Abt Associates Inc., Bethesda, Maryland, United States of America; 3 Health Finance and Governance Project, Abt Associates Inc., Bethesda, Maryland, United States of America

## Abstract

This paper is a country case study for the Universal Health Coverage Collection, organized by WHO. Abebe Alebachew and colleagues illustrate progress towards UHC and its monitoring and evaluation in Ethiopia.

*Please see later in the article for the Editors' Summary*

This paper is part of the PLOS Universal Health Coverage Collection. This is the summary of the Ethiopia country case study. The full paper is available as Supporting Information file Text S1.

## Background

This country case study is part of the Universal Health Coverage Collection. The World Health Organization defines universal health coverage (UHC) as a situation in which all people who need health services receive them, without incurring financial hardship [1]. UHC is currently perceived as a crucial component of sustainable development and listed as one of the possible goals of the post-2015 development agenda [1]–[3].

The objectives of this paper were to document the availability of globally proposed UHC indicators [4],[5] in Ethiopia; seek feedback from selected key informants on these indicators' relevance and feasibility [6]; review the country's overall capacity to collect and use UHC indicators; and compile existing estimates for proposed UHC indicators. The paper also aimed to inform the Ethiopian government as it develops its own UHC strategy and eventually implements such policies.

## Universal Health Coverage: The Policy Context

Ethiopia has not yet promulgated an official definition of UHC, although numerous strategies, policies, and guidelines are being implemented to achieve universal access to primary health care and reduce impoverishment due to health spending [7]–[13]. Existing strategies remain fragmented across health care services and financing mechanisms.

## Monitoring and Evaluation for UHC

Among the 61 proposed indicators [4],[5] that were explored in this paper to measure UHC, our review indicated that 28 are collected in Ethiopia through surveys and 14 are recorded and reported through the health management information system (HMIS) or other administrative sources. Twenty-seven indicators (44%) are not collected nor reported in any of the sources [14]–[24].

Ethiopia measures most of the major service coverage indicators related to reproductive, maternal, and child health and key infectious disease services using routine information systems and population-based surveys. However, indicators of chronic, non-communicable disease service coverage are generally not available. Three indirect measures of financial coverage are available from National Health Accounts estimations, but direct measures of catastrophic spending and impoverishment due to health care spending have not been calculated.

## Progress towards UHC in Ethiopia

**Figure 1:**
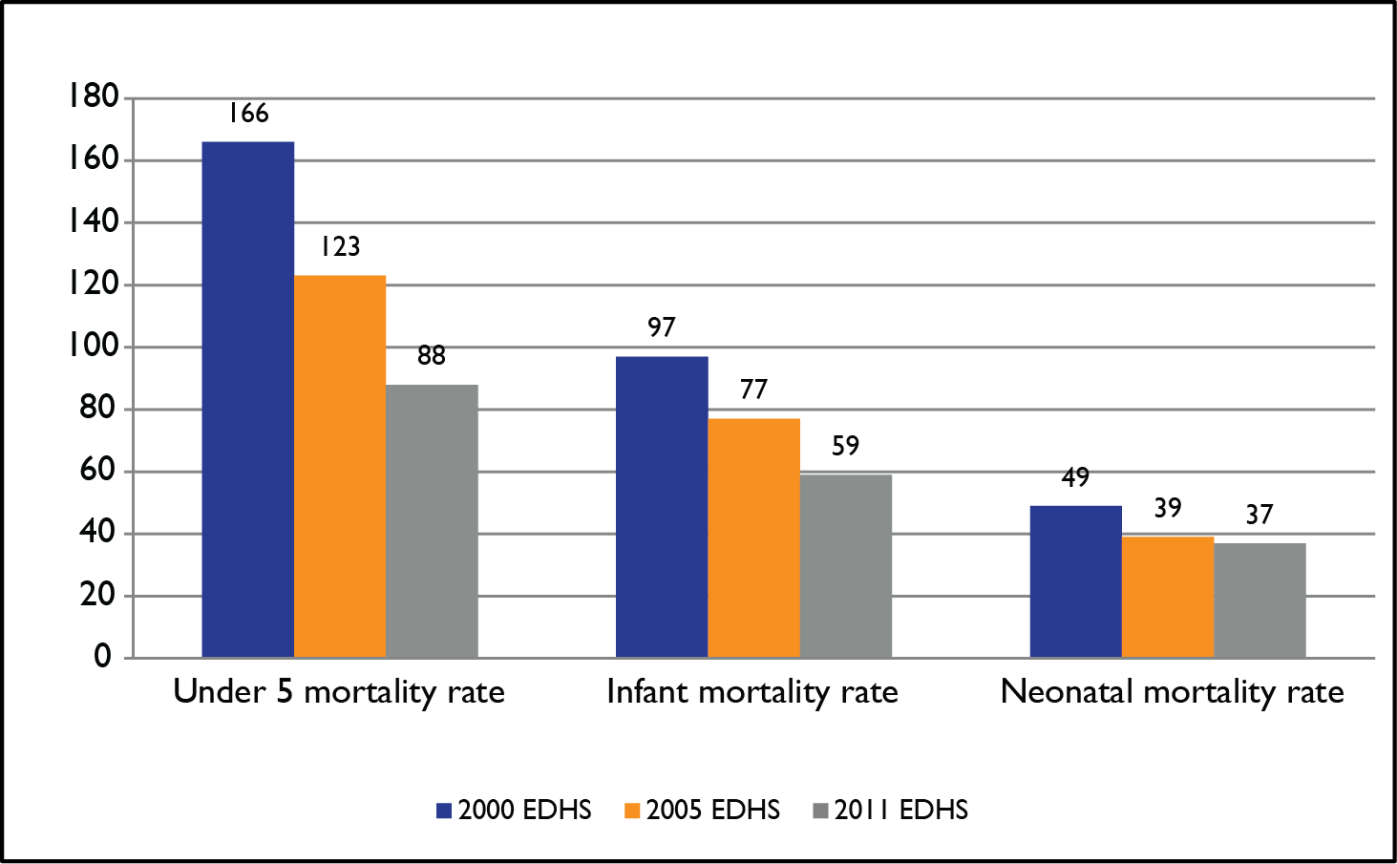
Trends in childhood mortality rates (deaths per 1,000 live births). Sources: [16]–[18].

Ethiopia has shown significant progress in reducing under-five, infant, and neonatal mortality rates over the last decade. These rates have declined by 47%, 39%, and 25%, respectively (Figure 1) [16]–[18]. According to the latest United Nations report, Ethiopia has achieved the Millennium Development Goal (MDG) goal of reducing child mortality well ahead of its 2015 deadline.

**Figure 2:**
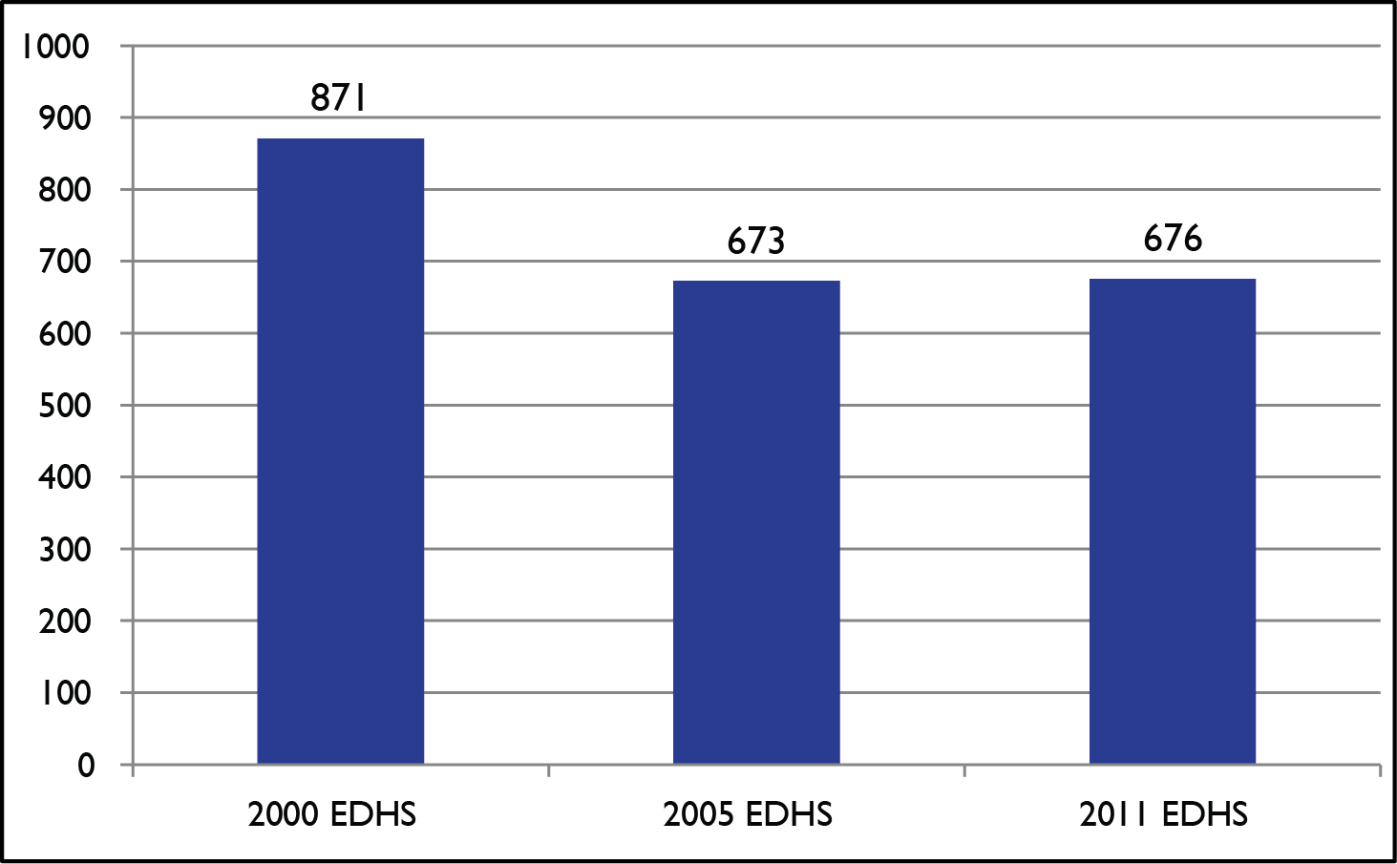
Trends in maternal mortality ratios (maternal deaths per 100,000 live births). Sources: [16]–[18].

Immunization rates and the delivery of other child health services have improved substantially since 2000 [14]–[18]. However, coverage trends have been mixed for maternal and reproductive health services; the maternal mortality ratio has registered no significant change since the 2005 Demographic and Health Survey (DHS) (Figure 2) [16]–[18].

The total number of health care facilities, particularly primary care centers, has increased nearly 10-fold since 2005 and distribution on a per capita basis is largely equitable; on the other hand, secondary and tertiary level service capacity has not improved significantly [14],[15]. This shortage has been one factor leading to socioeconomic disparities in the access to and utilization of hospital level health services; other factors include costs and geographical barriers [24]. The largest improvements in service coverage between 2005 and 2011 occurred among the wealthiest households [18],[24].

Ethiopia is moving to expand financial protection through various financing initiatives. A social health insurance law was recently passed and a national health insurance agency established. Pilot community-based health insurance schemes have also been initiated. Nonetheless, out-of-pocket spending remains a major source of health financing for Ethiopia's population and is a significant barrier for accessing and utilizing health services [19]–[22].

## Conclusions and Recommendations

Some of the WHO's proposed UHC measurement indicators may not yet be applicable or feasible in a low-income context like Ethiopia, particularly those requiring frequent, large population-based household surveys as well as those related to chronic conditions. Local stakeholders expressed a preference for indicators that are more programmatically relevant to their context and less resource-intensive to collect. If UHC is included in a post-MDG agenda, involving country representatives in selecting these indicators would harness political commitment towards UHC implementation.

Countries like Ethiopia should be assisted in defining and developing UHC strategies; technical support should also be given to build their capacity to collect, analyze, and use routine and survey-based information. Such capacity in Ethiopia is growing but still limited, and this has negatively impacted the quality and availability of data.

## Supporting Information

Text S1 The full country case study for Ethiopia.
(DOCX)

## Abbreviations

UHC: universal health coverage
