# Supplementary material for: Monitoring and Evaluating Progress towards Universal Health Coverage in Ethiopia
Source: PLoS Med. 2014 Sep 22;11(9):e1001696. doi: 10.1371/journal.pmed.1001696 (PMC4171462; doi:10.1371/journal.pmed.1001696)
Supplement: Text S1 — The full country case study for Ethiopia. (DOCX) [file pmed.1001696.s001.docx]

**Full Case Study: Monitoring and Evaluating Progress Towards Universal Health Coverage in Ethiopia**

Abebe Alebachew^1^, Laurel Hatt^2^ and Matthew Kukla^2^

^1^ Breakthrough International Consult, Addis Ababa, Ethiopia

^2^ International Health Division, Abt Associates Inc., Bethesda, MD, USA

*Corresponding author: Abebe Alebachew

Email: abebe.alebachew2008@gmail.com

**This paper is the full country case study to accompany the summary paper “Monitoring and Evaluating Progress Towards Universal Health Coverage in Ethiopia” that is part of the Universal Health Coverage Collection. Not commissioned; externally reviewed.**

**Abbreviations:**

EDHS = Ethiopian Demographic and Health Survey

EFY = Ethiopian Fiscal Year

EHIA = Ethiopian Health Insurance Agency

FMOH = Federal Ministry of Health

GOE = Government of Ethiopia

HMIS = Health Management Information System

MDG = Millennium Development Goal

NCD = Noncommunicable Disease

OOP = Out-of-pocket

UHC = Universal Health Coverage

WHO = World Health Organization

WMS = Welfare Monitoring Survey

**Abstract:** This paper reviews a list of WHO proposed UHC indicators and assesses their availability in a low-income country context. It specifically analyses the progress Ethiopia has made in achieving universal health coverage. The major findings of this study are as follows: (i) Of the proposed global indicators, most service coverage indicators are available in Ethiopia; (ii) Indicators for chronic conditions are not yet a priority or available in Ethiopia; (iii) While data for financial protection indicators are being collected in household income, consumption and expenditure surveys, many of these indicators are not yet available in country. As such, financial protection indicators do not yet allow for annual planning and programing. This paper recommends (a) for Ethiopia to develop a comprehensive definition and strategy for UHC including chronic conditions; (b) to redefine the proposed indicators within Ethiopia and other low-income countries' unique context; (c) to strengthen the capacity of low income countries so that they may generate the necessary information for monitoring the achievement of UHC.

**Summary Points:**

1. Ethiopia routinely measures most of the globally-proposed service coverage indicators for reproductive, maternal and child health and key infectious diseases. Indicators of chronic, non-communicable disease service coverage are generally not available. Some but not all of the proposed financial coverage indicators are available.
2. Some of the proposed UHC measurement indicators may not yet be applicable or feasible in a low-income context like Ethiopia, particularly those requiring frequent, large population-based household surveys as well as those related to chronic conditions. Local stakeholders expressed a preference for indicators that are more programmatically relevant to their context. If UHC is included in a post-MDG agenda, involving country representatives in selecting these indicators would harness political commitment towards UHC implementation.
3. Low-income countries will need support to build capacity to generate and utilize UHC measurement indicators. This might include technical assistance during the development of a UHC strategy or strengthening capacity to generate information on NCDs and financial risk protection.
4. Ethiopia has a functional health sector planning and monitoring system where relevant stakeholders at federal, regional and district (woreda) levels jointly assess health system performance. UHC performance review could be integrated in this process when a measurement framework is fully defined.

**1. Background**

Universal health coverage (UHC) as a goal of health policy development has gained wide acceptance at country and global levels since the publication of the World Health Report 2010 and is now seen as a critical component of sustainable development [1,2]. UHC has also been listed as one of the possible goals of the post-2015 development agenda [3]. Discussions on the suitability of UHC as a goal are often reduced to two questions: how should UHC be defined and how can it be measured and monitored? The World Health Organization (WHO) has defined UHC as a situation where all people who need health services receive them, without incurring financial hardship [1]. This definition entails two interrelated components: coverage with needed quality health services and access to financial risk protection, for everyone. The level and distribution of effective coverage of interventions and financial risk protection have been proposed as the focus of monitoring progress towards UHC [4].

Developing simple and sound measures to assess country, regional, and global situations and to monitor progress towards UHC is critical if this objective is to remain high on the global agenda and receive priority attention from country policymakers. While the basic definition of UHC is conceptually straightforward, developing feasible metrics of UHC is less so. Variations in countries’ epidemiology, health systems and financing, and levels of socioeconomic development imply different approaches to UHC implementation as well as a potential range of relevant metrics. Many countries working towards UHC already rely on locally-specific, routinely-collected service statistics to measure health system performance, and standard demographic, health and economic surveys contribute occasional snapshots of trends in health status measures and economic development. At the same time, establishing new global goals, indicators, and targets could have a critical impact on governments’ commitment to successful implementation of global declarations, such as the December 2012 United Nations Resolution making UHC a key global health objective.

While discussions on UHC measurement approaches have been occurring at the global level for a few years, less attention has been paid to country perspectives on this topic until recently. To advance discussion on the availability, feasibility, and relevance of various globally-proposed candidate indicators for UHC measurement – especially in resource-poor contexts – the Health Finance and Governance Project, funded by the United States Agency for International Development, conducted a case study in Ethiopia, a low-income country engaged in UHC efforts. The objectives of this study were to document the availability of proposed globally-proposed UHC indicators; seek feedback from key informants on these indicators’ relevance and feasibility; review the country’s overall capacity to collect and use UHC indicators; and compile existing estimates for proposed UHC indicators. The study also aimed to inform the Ethiopian government as it develops its own UHC strategy and eventually implements such policies.

This article summarizes the results of the case study. After a presentation of the case study methodology, we explore the indicators Ethiopia is already using to measure progress towards UHC. The subsequent sections evaluate Ethiopia’s capacity to collect data for and generate a set of proposed UHC indicators. We also issue recommendations for the government of Ethiopia and the international community based on the findings.

## Methodology

The research team compiled a list of indicators that are under consideration for global UHC monitoring from two primary sources: a WHO working paper by Evans et al. (2012) and an unpublished workshop report prepared as an output of a WHO- and Rockefeller Foundation-sponsored meeting in Bellagio in September 2012 [4, 5]. The list of 61 proposed indicators includes 52 tracer indicators of population service coverage and nine indicators of financial protection coverage. An additional set of 28 proxy health systems indicators was also reviewed.

The case study employed two methods: key informant interviews and secondary data analysis. Ten key informants representing the major stakeholders in Ethiopia’s UHC efforts (Table S1) were interviewed, including government, development partners, and implementing partners. The full list of research questions is published elsewhere.

| No. | Institution |
| --- | --- |
| 1 | Planning, Policy and M&E Directorate, Federal Ministry of Health (FMOH) |
| 2 | Resource Mobilization Directorate, FMOH |
| 3 | Medical Service Directorate, FMOH |
| 4 | Ethiopian Health Insurance Agency |
| 5 | WHO Ethiopia |
| 6 | United Nations Population Fund, Ethiopia |
| 7 | Italian Cooperation, Ethiopia |
| 8 | United States Agency for International Development Mission in Ethiopia |
| 10 | Abt Associates Inc., Ethiopia |

**Table S1:** Key Informant List

The scope of these data collection efforts was limited due to the constrained time period in which this research was undertaken (August–September 2013); we were unable to interview some key stakeholders, such as private sector associations and other donor agencies. The study team also obtained and analyzed relevant secondary data to assess availability of UHC indicators and to document trends over time. Data sources used in the analysis are presented in Table S2.

|  | Data Source | Type of data collection | Years of data used in the analysis | Approximate frequency of data collection |
| --- | --- | --- | --- | --- |
| 1 | Routine information system (health and health-related indicators) [7]; annual performance reports [8] | Routine health facility reports | 2000-2013 | Monthly/annually |
| 2 | Demographic and Health Survey [9-11] | Nationally representative household survey | 2000; 2005; 2011 | Every 5 years |
| 3 | National Health Accounts [12-15] | Surveys of donors, NGOs, government agencies, insurance companies, employers | 2000; 2004/05; 2007/8; 2011/12 | Every 3-4 years |
| 4 | Welfare Monitoring Survey [16] | Nationally representative household survey | 1996, 1998, 2000, 2004, 2011 | Irregular |
| 5 | Household income, consumption and expenditure survey [17] | Nationally representative household survey | 2011 | Every 4-5 years |

**Table S2:** Secondary data sources used in this study

**2. Universal health coverage: the policy context**

Ethiopia is a low-income country with a per capita gross domestic product (GDP) of US$513 in 2011/12 [18]. While the proportion of people living below the local poverty line has declined by roughly a third over the past decade, the fraction remains high at 28 percent [9]. Private final health consumption is estimated to constitute 3 percent of the GDP [18]. The country is federally structured and three tiers of government (federal, regional, and woreda (district)) allocate resources to the health sector.

Ethiopia has not yet promulgated an official definition of UHC (Ethiopia’s social health insurance strategy touches on UHC by defining the objective of a future social health insurance scheme as “…provide quality and sustainable universal health care coverage to the beneficiary through pooling of risks and reducing financial barriers at the point of service delivery” (FMOH 2010a)).

Nonetheless, the government of Ethiopia (GOE) is working on a vision for 2035, and, according to those interviewed for this study, the major policy imperative is expected to be UHC. UHC-designated reforms may be initiated in 2014 as part of the development of the Fifth Health Sector Development Program. As shown in Table S3, the country has various strategies aimed at improving access to a basic package of essential primary health care services and protecting users from catastrophic spending; many of the policies and strategies could fall under a “UHC” heading.

Such strategies and policies indicate that financial protection is of particular concern to the GOE. Estimates of the financial burden of out-of-pocket (OOP) spending for households range from 1.07 to 4 percent of household income [17,19]. To reduce the financial burden of user fees and premiums, the GOE has established various financial protection mechanisms. Table S3 presents each of these mechanisms and their associated challenges [20-22].

|  | Strategy | Definition | Main Challenges in Implementation |
| --- | --- | --- | --- |
| 1 | Exempted services | These services are free for all regardless of income. | Sustainability of financing for health commodities will become a challenge if and when donor resources reduced or withdrawn. |
| 2 | Essential services | The government subsidizes as much as 70% percent of non-medicine costs. Medicines are sold with a 25% mark-up. | Quality of services remains an issue. |
| 3 | Targeted fee waiver schemes for indigents | Local governments reimburse health providers for lost user fees when treating indigent patients. | Undercoverage of the very poor and wide regional variation in implementation. |
| 4 | Pilot community-based health insurance schemes | Government subsidizes 25% of the premiums of all members and the full premiums of the poor; district-level scheme managers are paid through government allocation. | Undercoverage of the poor; scalability of the schemes due to huge fiscal implications; and inadequate readiness of facilities to provide quality service. |
| 5 | Health insurance for the formal sector | The legal framework is in place, the Health Insurance Agency has been established, and some systems have been designed, but the agency has not yet started operations. | There is limited management capacity of the agency; limited readiness of facilities to provide quality care. |

**Table S3**: Strategies to Enhance Access and Financial Protection in Health

Data Sources: [20-22]

As these tables show, numerous separate strategies, policies, and guidelines are shaping Ethiopia’s efforts to provide universal primary health care in Ethiopia, addressing the UHC components of access, quality, and financial protection. However, they have yet to be consolidated into one coherent policy document with clearly articulated sources of financing. Moreover, although some isolated efforts have been initiated, there has been limited focus on noncommunicable diseases (NCDs).

**3. Monitoring and evaluation for UHC**

***Ethiopia’s Current and Proposed Systems for Measuring Progress towards UHC***

Ethiopia has an established monitoring and evaluation (M&E) system for its health sector, with an annual planning process that establishes targets and annual review meetings that assess performance [23]. There are 155 indicators in the current five-year Health Sector Development Program [24]; as indicated in Table S4, annual plans and performance reports capture 42 of these indicators on a yearly basis [19,24]. No direct financial protection indicators are planned for or monitored on annual basis. Thirty-six additional “key performance indicators” (KPIs) are used to monitor health service quality via a separate, parallel mechanism.

| Types of Indicators by Strategic Objectives | In HSDP IV (Five-year Plan) | Annual Plan | Comments |
| --- | --- | --- | --- |
| Access | 104 | 34 | Of the 104, 18 are impact indicators collected every five years only.* |
| Community ownership | 3 | 1 |  |
| Resource mobilization | 8 |  |  |
| Quality of health services | 9 | 1 | 36 additional KPI monitor the quality of hospital services. |
| Emergency preparedness and response | 1 | 1 |  |
| Pharmaceutical services | 7 | 1 |  |
| Regulatory system | 5 | 1 |  |
| Planning and M&E | 7 | 1 |  |
| Infrastructure | 8 | 1 |  |
| Human capital and leadership | 3 | 1 |  |
| Total | 155 | 42 | Of the 155, information on 44 are derived from surveys. |

**Table S4:** Types and Numbers of Health Sector Indicators Monitored in Ethiopia

Data Sources: [19,24]

***Comparison of Ethiopia’s UHC Measurement Approach with WHO-Proposed Indicators***

Ethiopia has three sources of health monitoring data relevant for UHC measurement: the Health Management Information System (HMIS), administrative reports, and surveys. Of the 61 WHO-proposed indicators that were explored in this study to measure UHC, our review indicated that 28 are collected in Ethiopia through surveys and 14 are recorded and reported through HMIS or other administrative sources. Twenty-seven indicators (44%) are not collected nor reported in any of the sources. Table S5 shows the availability of indicators, grouped by the major UHC elements of measurement.

| Elements of UHC Measurement | # of Indicators  Explored | # Available from surveys | # Available from routine sources | # Un-available |
| --- | --- | --- | --- | --- |
| Service coverage indicators | 52 | 27 | 12 | 21 |
| Financial protection indicators | 9 | 1 | 2 | 6 |

**Table S5:** Availability of proposed UHC indicators in Ethiopia’s information system

Data sources: [7-17]

Table S10 (end of file) provides a comprehensive list of these indicators, data collection methods, frequency and availability.

1. ***Service Coverage Indicators:***

Of the 52 indicators proposed for measuring service coverage, Ethiopia collects and uses 31 or roughly 60 percent (Table S6). All the proposed tracer indicators of maternity care, child nutrition, child vaccination, treatment of sick children, family planning, malaria, tuberculosis, and HIV/AIDS services are available. However, none of the proposed NCD or injury indicators (for coverage of cancer, cardiovascular disease, diabetes, chronic pain, chronic respiratory conditions, musculoskeletal conditions, mental health, vision, or dental services) are currently being collected. Key informants acknowledged concerns about an epidemiological transition in Ethiopia, including a rising burden of NCDs, but the priority given to these conditions remains marginal. Interviewees noted that there is a plan to undertake a Burden of Disease study in 2014 to estimate NCD prevalence rates and establish a baseline for future interventions.

| Service Coverage Indicators | # of Indicators  Explored | # Available from surveys | # Available from routine sources | # Un-available |
| --- | --- | --- | --- | --- |
| Maternal health | 5 | 5 | 3 | 0 |
| Child nutrition | 6 | 6 | 0 | 0 |
| Child vaccination | 5 | 5 | 3 | 0 |
| Treatment of sick children | 3 | 3 | 0 | 0 |
| Family planning | 2 | 2 | 1 | 0 |
| Malaria | 3 | 3 | 1 | 0 |
| Tuberculosis | 2 | 0 | 2 | 0 |
| HIV/AIDS | 4 | 2 | 2 | 0 |
| Cancer | 5 | 1 | 0 | 4 |
| Cardiovascular diseases | 8 | 0 | 0 | 8 |
| Diabetes | 1 | 0 | 0 | 1 |
| Chronic pain | 1 | 0 | 0 | 1 |
| Musculoskeletal conditions | 1 | 0 | 0 | 1 |
| Mental health | 1 | 0 | 0 | 1 |
| Vision care | 1 | 0 | 0 | 1 |
| Hearing care | 1 | 0 | 0 | 1 |
| Dental care | 1 | 0 | 0 | 1 |
| Other NCDs and injuries | 2 | 0 | 0 | 2 |
| Total | 52 | 27 | 12 | 21 |
|  |  |  |  | 40% |

**Table S6:** Availability of Proposed UHC Service Coverage Indicators

Data sources: [7-11]

Measurement of the quality of services, an element of “effective” coverage, is primarily limited to the tertiary level, where the 36 KPIs are currently tracked. All hospitals set annual quality targets based on these KPIs and report monthly on achievements. Perceived quality is tracked through quarterly patient satisfaction surveys. The focus of quality measurement to date has been on tracking system-wide progress (overall patient satisfaction, infection, and mortality rates) rather than disease- or service-specific indicators as in the WHO’s proposed measurement indicators. The only service-specific quality indicators being collected in Ethiopia are related to maternity and pediatric care. Efforts to assess quality of chronic disease services are just beginning. Given that Ethiopia’s vision for UHC is related to ensuring access to primary care, measures of quality outside health facilities (services provided through the health extension program for example) are rarely found.

1. ***Financial Protection Indicators***

In Ethiopia, 3 of the WHO’s 5 proposed “indirect” measures of financial protection – measures that usually correlate with but do not directly measure rates of impoverishment from health spending – are collected via routine government administrative reports or surveys (Table S7) (These indirect measures include OOP payments as a percent of total health spending; government health spending as a percent of GDP; government health spending as a percentage of total government spending; etc.). None of the 4 “direct” financial protection indicators, measuring the financial burden of health spending to families, is calculated (These direct measures include incidence and depth of catastrophic health spending and medical impoverishment). National Health Accounts estimations and Household Income, Consumption, and Expenditure surveys are conducted on a regular basis; however, final survey reports show only the overall percentage of total household income spent OOP on health care (about 1 percent in 2011). The reports do not analyze the percentage of households that are impoverished as a result of OOP spending. Thus, while efforts are being made reduce the burden of OOP spending on households, these strategies are not yet reflected in the official indicator list for health sector performance.

| Financial Coverage Indicators | # of Indicators  Explored | # Available from surveys | # Available from routine sources | # Un-available |
| --- | --- | --- | --- | --- |
| Direct | **4** | **0** | **0** | **4** |
| Indirect | **5** | **1** | **2** | **2** |
| Total | **9** | **1** | **2** | **6** |
|  |  |  |  | **67%** |

**Table S7:** Availability of Proposed UHC Financial Protection Indicators

Data sources: [12-17]

The 2013/14 Annual Plan of the new Ethiopian Health Insurance Agency (EHIA) introduced financial protection-related performance indicators. The proposed indicators include coverage of risk pooling schemes, percentage of the poor whose premium is paid by government, and percentage of risk pooling scheme members utilizing services. While not direct measures of financial protection, these three indicators seem programmatically relevant for measuring annual efforts made to reduce the number of people paying out-of-pocket and to support the very poor. Other EHIA indicators will reflect the quality of services provided to members of the risk pooling schemes and patient satisfaction. The proposed “direct” financial protection indicators listed above were perceived by key informants to be important impact indicators, but less programmatically relevant in the Ethiopian context.

1. ***Additional Health System Performance Indicators***

Table S8 presents several WHO-recommended additional indicators that could be used as proxies for UHC by measuring health system performance. The detailed definitions of these indicators in the Ethiopian context are listed in Table S10. They include measurements of health workforce and infrastructure, service readiness, quality of care, and health outcomes. Roughly 54 percent of these additional proposed indicators are available in Ethiopia, with notable gaps in the availability of service readiness and quality indicators. The Service Provision Assessment survey that will be conducted in EFY 2013/14 is expected to collect some of these indicators.

| Additional Indicators | # of Indicators  Explored | # Available from surveys | # Available from routine sources | # Un-available |
| --- | --- | --- | --- | --- |
| Health financing | 1 | 1 | 0 | 0 |
| Health workforce | 2 | 0 | 2 | 0 |
| Infrastructure | 2 | 0 | 2 | 0 |
| Vital Registration | 2 | 0 | 0 | 2[ |
| Service access and readiness | 4 | 1 | 1 | 3 |
| Service quality and safety | 4 | 0 | 0 | 4 |
| Risk and behavioral factors | 4 | 1 | 2 | 1 |
| Health status | 8 | 5 | 0 | 3 |
| Responsiveness | 1 | 1 | 0 | 0 |
| Total | 28 | 9 | 7 | 13 |
|  |  |  |  | 46% |

**Table S8:** Availability of Proposed Additional UHC Indicators

Data sources: [7-17]

**4. Progress towards UHC in Ethiopia**

This section provides a snapshot of Ethiopia’s progress towards UHC to provide a sense for how the country might monitor its UHC efforts. We use available indicators of health outcomes, service coverage, and financial coverage in Ethiopia. While mortality indicators – notably the Millennium Development Goals (MDG) and high-level impact targets – are not synonymous with coverage, they provide a snapshot of how health system performance has changed over a 12-year period.

Ethiopia has shown significant progress in reducing under-five, infant, and neonatal mortality rates over the last decade. These rates have declined by 47, 39, and 25 percent, respectively (see Figure S1) [9-11]. According to the latest United Nations report, Ethiopia achieved the MDG goal of reducing child mortality well ahead of 2015. Many of the child health services seem to have witnessed significant improvement in reaching their target populations (see Figure S2) [8]: since 2000, coverage of DPT3 has doubled; immunization against measles has increased by 150 percent and full immunization has increased by 230 percent, according to routine information systems. However, reported coverage rates vary significantly between population-based surveys and routine information systems.

**Figure S1:** Trends in Childhood Mortality Rates (deaths per 1,000 live births)

Data Sources: [9-11].

Note: EDHS = Ethiopian Demographic and Health Survey

**Figure S3:** Trends in Coverage of Immunizations (%)

Data Source: [8]

For maternal and reproductive health services, coverage trends have been mixed. Access to pre- and postnatal care and family planning has increased, but rates of skilled attendance at delivery – the major determinant for reducing the maternal mortality ratio – have shown only a very marginal increase since 2008, according to routine data sources. As shown in Figure S2, survey-based estimates of the maternal mortality ratio also showed no change from the 2005 DHS (673 maternal deaths per 100,000 live births) to the 2011 DHS [9,11].

**Figure S2:** Trends in Maternal Mortality Ratios (maternal deaths per 100,000 live births)

Data Source: [9-11]

One of Ethiopia’s key priorities over the past decade has been to increase geographic access to care as measured by the number of primary health care facilities. Over 15,000 health posts and 2,780 health centers have been constructed since 2005, and the total number of health facilities nationwide (including those owned by the private sector) has increased more than tenfold. This investment has dramatically increased the reach of primary health care services (Figure S4) [8]. According to the WMS 2012, 65 percent (84 percent) of households are within five (ten) kilometers of the nearest health post, and 38 percent (60 percent) within five (ten) kilometers of a health center.

**Figure S4:** Trends in Coverage of Maternal and Reproductive Health Services **(%)**

Data Source: [8]

Note: CAR stands for Contraceptive Acceptance Rate; this is tracked via the HMIS and is used to proxy contraceptive prevalence rate.

Expansion of hospital-level services has taken place at a slower pace, as per Figure S5 [7,8]. From 2000 to 2011, while the number of hospitals increased from 103 to 212, hospital inpatient bed availability stagnated at around 2.1 per 10,000 due to population growth. However, with the government aiming to expand comprehensive obstetric care services, there is growing pressure to expand the number of primary hospitals to more than 800 (over one per district) over the next three years [24]. Currently, 14 percent of households live within five kilometers of a hospital, and 21 percent within ten kilometers.

**Figure S5:** Trends in the Total Number of Health Facilities

Data Source: [8]

The regional distribution of health facilities on a per capita basis is largely equitable, according to government reports. However, urban-rural disparities in the distribution of health facilities are significant. In urban areas, roughly 88 percent of households live within five kilometers of a primary health service provider, and nearly half of urban households are within five kilometers of a hospital. The corresponding proportion of rural households with this geographic access is 63 percent (health post), 24 percent (health center), and 1.5 percent (hospital) [16].

Routine data sources and population-based surveys tell different stories about whether Ethiopians have been seeking outpatient care more frequently since 2000. Routine services statistics indicate that despite the expansion of primary health care facilities, outpatient visits per 10,000 population have increased only by about 14 percentage points. Data from population surveys on the other hand show a larger 22 percentage point increase in outpatient visits. There is again a wide difference in utilization between urban and rural areas (see Figure S6) [16].

**Figure S6:** Trends in the Number of Outpatient Department Visits for Curative Care per 10,000 People per Year

Data Source: [16]

There are also socioeconomic disparities in utilization of curative care. The WMS 2012 found that for the 30 percent of respondents who did not seek care for a recent illness, their major reported barrier was the cost of care. Analysis of utilization of selected fee-exempted services shows that the poorest quintile is far behind in using these services (see Table S9) for reasons related to social and cultural factors as well as cost and geographic access [16]. Despite progress made in expanding primary care to rural areas across all regions and woredas, the largest improvements in service coverage between 2005 and 2011 occurred among the wealthiest households.

| Coverage Indicators | Wealth Quintile | 2005 | 2011 |
| --- | --- | --- | --- |
| Skilled attendance at delivery | **Poorest quintile** | 1% | 2% |
|  | **Richest quintile** | 27% | 46% |
| Postnatal care | **Poorest quintile** | 1% | 4% |
|  | **Richest quintile** | 24% | 31% |
| DPT3 | **Poorest quintile** | 26% | 26% |
|  | **Richest quintile** | 48% | 62% |
| Contraceptive Prevalence Rate | **Poorest quintile** | 4% | 13% |
|  | **Richest quintile** | 37% | 52% |
| Unmet Need for Family Planning | **Poorest quintile** | 33% | 31% |
|  | **Richest quintile** | 24% | 15% |

**Table S9:** Coverage Rates for Selected Indicators in the Poorest and Richest Wealth Quintiles

Data sources: [11,16]

Ethiopia is moving to expand financial protection through various financing initiatives, as described in the background section. A social health insurance strategy law was passed and the Health Insurance Agency has been established, though it has yet to start operations. Pilot community-based health insurance schemes have been initiated in 13 districts, so far enrolling about 50% of households in those areas. Nonetheless, OOP spending remains one of the major sources of health financing for the population, and one of the possible deterrents to use (see Figure S7) [12-15,25].

**Figure S7:** Trends in OOP Spending as a Share of Total Health Expenditure (%)

Data Source: [12-15]

* A methodological revision in estimating OOP spending in the 2007/08 National Health Accounts may be the reason for the increase in OOP spending as a percentage of total health sector spending that year. Specifically, the 2007/08 OOP spending estimate is based on an independent household health expenditure survey while prior estimates were based on Central Statistical Agency-generated estimates.

** Preliminary data

**5. Conclusions and recommendations**

**Local Capacity to Collect, Analyze, and Use UHC Indicators**

In addition to the question of whether Ethiopia’s existing systems include relevant indicators for UHC measurement, strengths and weaknesses in the HIS more broadly will influence Ethiopia’s capacity to generate these measures. Our review of secondary data and key informant interviews highlighted concerns about the quality of existing systems. Weaknesses in basic infrastructure (Internet, electricity, and hardware), especially at facility and woreda levels, present challenges for both survey-based and routine data collection. While there is general consensus that surveys such as the DHS are of high quality – both in terms of their validity and reliability – their ability to accurately monitor changes in self-reported health care coverage are limited by low literacy rates, recall bias (especially for long-term recall of detailed information like vaccine doses), and survey fatigue. Survey estimates of income and consumption, including health expenditures, are also subject to well-known limitations [26].

The quality of Ethiopia‘s routine information system is mixed, as in most low- and middle-income countries, and efforts to strengthen and scale up the electronic HMIS and other vertical information systems are ongoing. A recent annual data quality assessment in Ethiopia noted weaknesses such as under- and over-reporting (and lack of accountability for accurate reporting), challenges with timeliness and quality of data, and inadequate supportive supervision; most M&E staff at health facilities did not have adequate knowledge of reporting procedures or indicator calculation [27]. The quality of routine data can only be improved when it is used for planning and monitoring at local levels, yet only a third of facilities compared their plans with performance every quarter. While the GOE has tried to instill a culture of information use through its annual planning process, there is still work to be done.

**Overall Recommendations on the Selection of Global UHC Indicators**

To gain country-level commitment, UHC measurement indicators need to be technically sound, programmatically useful, and politically valued by country stakeholders. Key informants interviewed for this study suggested that if UHC is to become part of a post-2015 agenda and guide policy decisions, a smaller number of indicators should be tracked. They argued that the indicators for UHC should focus on a few impact, outcome, and health systems indicators rather than a list of many disease-specific indicators. Proposed global indicators should be further reviewed for the availability of data, relevance for local policy and programming, and cost of data collection. There must also be ownership and consensus on the list of indicators by all stakeholders, particularly health programs within countries. Some of the proposed financial protection indicators may be useful to understand whether the poor are being impoverished by health care costs, but they require costly surveys and as such may not be feasible for routine monitoring.

Below are several additional recommendations that the authors suggest:

- Ethiopia should work to consolidate its strategy for UHC and UHC measurement: Consolidating the various separate service delivery and financing strategies and plans into one UHC strategy for the 2015–2020 period would help to ensure a consistent vision and promote buy-in by policymakers at the federal, regional, and woreda levels. Developing a consolidated plan, sharing the plan with stakeholders, building awareness of what UHC means and how it will be implemented – and how it will be measured – will build momentum for this critical goal.
- The global community, and individual countries, should select a concise list of programmatically relevant indicators for systematic monitoring of UHC reforms: The list of proposed global indicators reviewed was very long and may not get buy-in at the country level. Fewer tracer indicators that balance the assessment of health system performance with more distal coverage outcomes are recommended. Selection of indicators at the country level should be guided by local programmatic relevance, priority health burdens (such as communicable vs. noncommunicable diseases), and the ability to mobilize political commitment. Both in Ethiopia and globally, there is need for better articulation of financial protection indicators that can be gathered routinely and used for annual planning and performance monitoring.
- Strengthen capacity for collecting, estimating, and sharing UHC indicators: Effective progress towards UHC will require good policy analysis capacity to inform strategy development as well as programming. Low- and middle-income countries, including Ethiopia, could benefit from technical assistance in the development of relevant UHC strategies. Poor countries just entering the epidemiological transition and beginning to establish risk pooling schemes are particularly in need of capacity investments in their routine information systems in order to generate NCD and financial protection indicators. In Ethiopia, the Federal Ministry of Health must proactively engage with the Central Statistics Agency when health information and welfare monitoring surveys are planned to ensure that UHC-relevant information is collected, analyzed, and disseminated. Finally, it would be useful to set up a technical group of epidemiologists, demographers, statisticians, and health economists from different UHC stakeholder institutions in Ethiopia to analyze health information system challenges and disparities in results gathered from routine and survey findings. Major capacity issues must be addressed systematically to ensure greater concurrence between the routine and survey results and improve the quality of available UHC monitoring data.

| **Box 1: Recommendations**   1. Globally-proposed UHC measurement indicators should be further reviewed in consultation with countries, to ensure that they are programmatically relevant for health sector progress monitoring and to harness political commitment from decision makers. 2. Low-income countries like Ethiopia should be assisted in defining and developing UHC strategies; technical support should also be given to build countries’ capacity to collect, analyze and use routine and survey-based information. 3. Ethiopia may wish to give priority to measuring NCD indicators given that an epidemiological transition is taking place. This could be initiated by developing a comprehensive NCD strategy. |
| --- |

**References**

[1] World Health Organization (2010) The World Health Report: Health Systems Financing: the Path to Universal Coverage. Geneva: World Health Organization.

[2] Brearly L, Marten R, O’Connell T (2013) Universal Health Coverage: A Commitment to Close the Gap. New York: Rockefeller Foundation, Save the Children, the United Nations Children’s Fund (UNICEF) and the World Health Organization.

[3] Vega J (2013) Universal health coverage: the post-2015 development agenda. New York: Rockefeller Foundation.

[4] Evans D, Saksena P, Elovainio R, Boerma T (2012) Measuring Progress towards Universal Coverage. Working paper. Geneva: World Health Organization.

[5] World Health Organization (2012) Measurement of trends and equity in coverage of health interventions in the context of universal health coverage. Unpublished workshop summary report, Bellagio, September 17–21.

[6] Alebachew A, Hatt L, Kukla M, Nakhimovsky S (2014) Universal Health Coverage Measurement in a Low-Income Context: An Ethiopian Case Study. Bethesda, MD: Health Finance & Governance Project, Abt Associates Inc. Available: <http://www.hfgproject.org/wp-content/uploads/2014/04/Ethiopia_Case-Study-on-Measuring-Progress-towards-UHC_Final.pdf>

[7] Federal Ministry of Health (Ethiopia). Various. Health and health related indicator reports (EFY 2000-2013).

[8] Federal Ministry of Health (Ethiopia). Various. Health management information system reports (EFY 2000-2013).

[9] Central Statistical Agency (Ethiopia) and ICF International (2012) Ethiopia Demographic and Health Survey 2011. Addis Ababa, Ethiopia and Calverton, Maryland, USA. Available: <http://www.measuredhs.com/pubs/pdf/FR255/FR255.pdf>

[10] Central Statistical Agency (Ethiopia) and ORC Macro (2001) Ethiopia Demographic and Health Survey 2000. Addis Ababa, Ethiopia and Calverton, Maryland, USA.

[11] Central Statistical Agency (Ethiopia) and ORC Macro (2006) Ethiopia Demographic and Health Survey 2005. Addis Ababa, Ethiopia and Calverton, Maryland, USA. Available: <http://www.measuredhs.com/pubs/pdf/FR179/FR179%5B23June2011%5D.pdf>

[12] Federal Ministry of Health (Ethiopia) (2013) Ethiopia’s Fifth National Health Accounts, 2010/11 [DRAFT]. Addis Ababa, Ethiopia.

[13] Federal Ministry of Health (Ethiopia) Health Care Financing Team, Planning and Programming Directorate (2010) Ethiopia’s Fourth National Health Accounts, 2007/08. Addis Ababa, Ethiopia. Available: <http://www.who.int/nha/country/eth/ethiopia_nha_4.pdf>

[14] Federal Ministry of Health (Ethiopia) Health Care Financing Team, Planning and Programming Directorate (2006) Ethiopia’s Third National Health Accounts 2004/05. Bethesda, MD: Partners for Health Reform*plus* Project, Abt Associates Inc. Available: <http://www.who.int/nha/country/Ethiopia-NHA3.pdf>

[15] Federal Ministry of Health (Ethiopia) Health Care Financing Secretariat (2003) Ethiopia’s Second National Health Accounts 1999/2000. Addis Ababa, Ethiopia. Available: <http://www.ethiopianreview.com/pdf/001/NHA2.>pdf

[16] Central Statistics Agency (Ethiopia) (1997, 2000, 2005, 2012) Welfare Monitoring Surveys. Addis Ababa, Ethiopia.

[17] Central Statistical Agency (Ethiopia) (2011) Household, Income, Expenditure and Consumption Survey. Addis Ababa, Ethiopia.

[18] Ministry of Finance and Economic Development (Ethiopia) (2013) Estimates of the 2010/11 Base Year Series (EFY 2003). Addis Ababa, Ethiopia.

[19] Federal Ministry of Health (Ethiopia) (2013) Health Sector Development Plan IV Midterm Review Report. Addis Ababa, Ethiopia.

[20] Harvard School of Public Health (2013) Rapid diagnostic study on resource tracking in Ethiopia.

[21] Federal Ministry of Health (Ethiopia) (2010) User fee revision in Ethiopia. Addis Ababa, Ethiopia.

[22] Purvis G, Alebachew A, Wendwossen F (2011) Health Sector Financing Reform Midterm Project Evaluation. Washington, DC: Global Health Technical Assistance Project.

[23] Altman D, Alebachew A, Vogus A, Silla T, Won A (2012) Assessment of Evidence-based Planning in Ethiopia. Bethesda, MD: Health Systems 20/20 project, Abt Associates Inc.

[24] Federal Ministry of Health (Ethiopia) (2010) Health Sector Development Program IV 2010/11-2014/15. Addis Ababa, Ethiopia.

[25] Federal Ministry of Health (Ethiopia) (2010) Health Insurance Strategy. Addis Ababa, Ethiopia.

[26] Deaton, A (1997) The Analysis of Household Surveys: A micro-econometric approach to development policy. Washington, DC: World Bank.

[27] Federal Ministry of Health (Ethiopia) (2012) Preliminary Report on Data Quality Assessment. Addis Ababa, Ethiopia.

| **Category** | **Indicator** | **Data Collection Method** | **Frequency** | **Publication Time Lag** |
| --- | --- | --- | --- | --- |
| **Health Service Coverage** | |  |  |  |
| **Maternity Care** | Antenatal care: 4 or more visits | National household surveys | Every 5 years | 12 to 18 months |
|  | Antenatal care: Any visits | Routine HMIS; National household surveys | Monthly; Every 5 years | 2-3 months; 12 to 18 months |
|  | Skilled birth attendance | Routine HMIS; National household surveys | Monthly; Every 5 years | 2-3 months; 12 to 18 months |
|  | Institutional delivery | National household surveys | Every 5 years | 12 to 18 months |
|  | Postnatal care | Routine HMIS; National household surveys | Monthly; Every 5 years | 2-3 months; 12 to 18 months |
| **Child Nutrition** | Coverage of exclusive breastfeeding | National household surveys | Every 5 years | 12 to 18 months |
|  | Children under 5 who are stunted | National household surveys | Every 5 years | 12 to 18 months |
|  | Children under 5 who are underweight | National household surveys | Every 5 years | 12 to 18 months |
|  | Children under 5 who are overweight | National household surveys | Every 5 years | 12 to 18 months |
|  | Children under 5 who are wasted | National household surveys | Every 5 years | 12 to 18 months |
|  | Low birth weight among newborns | National household surveys | Every 5 years | 12 to 18 months |
| **Child Vaccination** | DPT3/pentavalent vaccine | Routine HMIS; National household surveys | Monthly; Every 5 years | 2-3 months; 12 to 18 months |
|  | Measles | Routine HMIS; National household surveys | Monthly; Every 5 years | 2-3 months; 12 to 18 months |
|  | BCG | National household surveys | Every 5 years | 12 to 18 months |
|  | Polio | National household surveys | Every 5 years | 12 to 18 months |
|  | Hepatitis B | (included in pentavalent vaccine–see above) | Monthly; Every 5 years | 2-3 months; 12 to 18 months |
| **Treatment of Sick Children** | Suspected pneumonia taken to health facility | National household surveys | Every 5 years | 12 to 18 months |
|  | Suspected pneumonia treated with antibiotics | National household surveys | Every 5 years | 12 to 18 months |
|  | Diarrhea treated with oral rehydration therapy | National household surveys | Every 5 years | 12 to 18 months |
| **Family Planning** | Unmet need for family planning | National household surveys | Every 5 years | 12 to 18 months |
|  | Contraceptive use rate | Routine HMIS; National household surveys | Monthly; Every 5 years | 2-3 months; 12 to 18 months |
| **Malaria** | Children sleeping under insecticide-treated net | National household surveys | Every 5 years | 12 to 18 months |
|  | Fever treated with antimalarials/ACT | National household surveys | Every 5 years | 12 to 18 months |
|  | Households with indoor residual spraying | Routine HMIS; National household surveys | Monthly; Every 5 years | 2-3 months; 12 to 18 months |
| **Tuberculosis** | TB case detection rate | Routine Program Quarterly reports | Quarterly | 2-3 months |
|  | Treatment success rate among estimated cases | Routine HMIS | Quarterly | 2-3 months |
| **HIV/AIDS prevention/treatment** | Condom use at higher risk sex (age 15-24) | National household surveys | Every 5 years | 12 to 18 months |
|  | ARV therapy among those in need | Routine HMIS | Monthly | 2-3 months |
|  | ARV prophylaxis among HIV+ pregnant women | Routine HMIS | Monthly | 2-3 months |
|  | PMTCT among HIV+ women | *N/A* | *N/A* | *N/A* |
|  | Male circumcision rate | National household surveys | Every 5 years | 12 to 18 months |
| **Cancer** | HPV vaccination | N/A | N/A | N/A |
|  | Tobacco use | National household surveys | Every 5 years | 12 to 18 months |
|  | Cervical cancer screening (20-64 years) | N/A | N/A | N/A |
|  | 5-year survival rate (specific cancers) | N/A | N/A | N/A |
|  | Mammography screening rate | N/A | N/A | N/A |
| **Cardiovascular Diseases** | Hypertension prevalence | Research (cross-sectional studies) | Irregular | N/A |
|  | Salt intake | N/A | N/A | N/A |
|  | Hypertension control follow up visit | N/A | N/A | N/A |
|  | Acute Myocardial Infarction survival | N/A | N/A | N/A |
|  | Physical activity measures | N/A | N/A | N/A |
|  | Hypertension treatment coverage | N/A | N/A | N/A |
|  | Angina treatment coverage | N/A | N/A | N/A |
|  | Cardiovascular disease preventive drug therapy for higher risk groups | N/A | N/A | N/A |
| **Diabetes** | Diabetes treatment coverage | N/A | N/A | N/A |
| **Chronic Pain** | Coverage of pain relief for those with chronic pain | N/A | N/A | N/A |
| **Chronic Respiratory Conditions** | Asthma/Chronic Obstructive Pulmonary Disease treatment coverage | N/A | N/A | N/A |
| **Musculoskeletal Conditions** | Arthritis treatment coverage | N/A | N/A | N/A |
| **Mental Health** | Depression treatment coverage | N/A | N/A | N/A |
| **Vision Care** | Spectacle coverage | N/A | N/A | N/A |
|  | Cataract surgery coverage | N/A | N/A | N/A |
| **Hearing Care** | Hearing aid coverage | N/A | N/A | N/A |
| **Dental Care** | Dental care coverage | N/A | N/A | N/A |
| **Other Non-Communicable Diseases** | Asthma (re) admission rate | N/A | N/A | N/A |
|  | Smoking cessation rate | N/A | N/A | N/A |
| **Injuries** | Coverage with rapid emergency response | N/A | N/A | N/A |
| **Financial Coverage** | |  |  |  |
| **Direct** | Incidence of catastrophic health expenditure due to out-of-pocket payments | N/A | N/A | N/A |
|  | Mean positive overshoot of catastrophic payments | N/A | N/A | N/A |
|  | Incidence of impoverishment due to out-of-pocket payments | N/A | N/A | N/A |
|  | Poverty gap due to out-of-pocket payments | N/A | N/A | N/A |
| **Indirect** | Out-of-pocket payments as a share of total health expenditure | National Health Accounts | Every 3-4 years | 6 months |
|  | Legal entitlement to health services through insurance or direct government funding/provision | N/A | N/A | N/A |
|  | Government health expenditure as a share of GDP | Routine Administrative reports; National Accounts | Annually |  |
|  | Government health expenditure as a share of general government expenditure | Routine Administrative reports | Annually | 2-3 months |
|  | Median price of generic drugs compared to international reference pricing | N/A | N/A | N/A |
| **Additional Possible Indicators** | |  |  |  |
| **Health financing** | Total health expenditure per capita | National Health Accounts | Every 3-4 years | 6 months |
| **Health workforce** | Number of health workers (specialists, general practitioners, health officers, nurses, midwives) per 10,000 population | Routine HMIS | Annually | 2-3 months |
|  | Annual number of graduates (doctors, health officers, nurses, midwives) of health profession educational institutions per 100,000 population | Routine Administrative reports | Annually | 2-3 months |
| **Infrastructure** | Number of hospitals per 10 000 population | Routine HMIS | Annually | 2-3 months |
|  | Number of health centers per 10 000 population | Routine HMIS | Annually | 2-3 months |
|  | Number of Health Posts per 10 000 population | Routine HMIS | Annually | 2-3 months |
|  | Hospital beds per 10,000 population | Routine HMIS | Annually | 2-3 months |
| **Vital Registration** | Percent of deaths that are registered | N/A | N/A | N/A |
|  | Percent of births registered | N/A | N/A | N/A |
| **Service access and readiness** | General service readiness index (from SARA or other assessment tool) or service-specific readiness indicators | N/A | N/A | N/A |
|  | Average availability of 14 selected medicines (using WHO/HAI tool) | N/A | N/A | N/A |
|  | Median consumer price ratio for tracer medicines (using WHO/HAI tool) | N/A | N/A | N/A |
|  | Number and mean outpatient visits per person per year | Routine HMIS; National household surveys | Monthly; every 5 years | 2-3 months; 12 to 18 months |
| **Service quality and safety** | 30-day hospital case fatality rate from acute myocardial infarction | N/A | N/A | N/A |
|  | 30-day hospital case fatality rate from stroke | N/A | N/A | N/A |
|  | Waiting time to elective surgeries (cataract, PTCA [angioplasty], hip replacement) | N/A | N/A | N/A |
|  | Surgical wound infection rate (% of all surgical interventions) | N/A | N/A | N/A |
| **Risk factors and behaviors** | Alcohol per capita consumption (per drinker) | N/A | N/A | N/A |
|  | Obesity among adults | National household surveys | Every 5 years | 12 -18 months |
|  | Access to safe water | Routine Administrative reports | Annually | 2-3 months |
|  | Access to improved sanitation | Routine Administrative reports | Annually | 2-3 months |
| **Health Status** | Life expectancy at birth | Global estimate | not known | N/A |
|  | Childhood mortality rates (underfive, infant, neonatal, perinatal mortality) | National household surveys | Every 5 years | 12 to 18 months |
|  | Maternal mortality ratio | National household surveys | Every 5 years | 12 to 18 months |
|  | TB prevalence | N/A | N/A | N/A |
|  | HIV prevalence among young people (15-24) | National household surveys; sentinel surveillance | Every 4-5 years; annually | 12 to 18 months; 2-3 months |
|  | Incidence of measles | N/A | N/A | N/A |
|  | Incidence of neonatal tetanus | N/A | N/A | N/A |
|  | Adolescent fertility rate | National household surveys | Every 5 years | 12 to 18 months |
| **Responsiveness** | User satisfaction with health services | Occasional surveys | Irregular | N/A |

**Table S10**: Data collection methods, frequency, and publication time lag for proposed UHC monitoring indicators in Ethiopia

Data sources: [7-17]
